# Reply to “Reassessing the Proposed Creatine–PrP Axis in Endometriosis: Methodological and Mechanistic Considerations”

**DOI:** 10.1002/advs.76708

**Published:** 2026-07-20

**Authors:** Siman Chen, Xiaoqian Ma, Yukai Liu, Zhiqi Zhong, Chunyan Wei, Mingqing Li, Xiaoyong Zhu

**Affiliations:** ^1^ Obstetrics & Gynecology Hospital of Fudan University Shanghai Key Lab of Reproduction and Development Shanghai Key Lab of Female Reproductive Endocrine Related Diseases Fudan University Shanghai People's Republic of China; ^2^ Fujian Provincial Key Laboratory of Reproductive Health Research Department of Obstetrics and Gynecology The First Affiliated Hospital of Xiamen University, School of Medicine Xiamen University Fujian People's Republic of China; ^3^ Xinglin College Nantong University Nantong People's Republic of China; ^4^ Department of Reproductive Immunology The International Peace Maternity and Child Health Hospital School of Medicine Shanghai Jiao Tong University Shanghai People's Republic of China; ^5^ Shanghai Key Laboratory of Embryo Original Diseases Shanghai People's Republic of China

**Keywords:** creatine, endometriosis, peritoneal fluid

## Abstract

This Correspondence is a formal response to the Comment by Dr. Machado regarding our publication “Creatine Promotes Endometriosis by Inducing Ferroptosis Resistance via Suppression of PrP.” We address several conceptual concerns raised in the Comment and clarify the physiological relevance of creatine concentrations, the methodological framework for PrP target identification, and the interpretation of human cohort data. We further provide evidence supporting creatine accumulation in endometriotic lesions and peritoneal fluid, and clarify the experimental basis for PrP target identification using integrated biochemical, computational, and functional analyses. Rather than demonstrating a direct molecular interaction, our findings instead provide convergent evidence consistent with PrP acting as a candidate functional target of creatine. We also reinforce the association between creatine exposure, ferroptosis resistance, and lesion progression, while acknowledging that definitive biochemical validation of direct binding remains to be established. Importantly, our study provides translational support for mechanistic investigations in endometriosis, rather than establishing a definitive clinical biomarker. Collectively, we highlight the metabolic significance of creatine–PrP signaling in endometriosis and discuss future directions for mechanistic and clinical validation.

## 1 Physiological Relevance of Creatine Concentrations

We sincerely thank Dr. Machado for his interest in our work and for the thoughtful comments regarding our article, ‘Creatine Promotes Endometriosis by Inducing Ferroptosis Resistance via Suppression of PrP.’ We appreciate the opportunity to clarify several methodological and conceptual points raised in the Comment. Constructive discussion is essential for advancing scientific understanding, and we are pleased to address these issues below.

The Comment raises the concern that the creatine concentrations used in our in vitro and in vivo experiments may exceed physiological levels. We agree that the physiological relevance of metabolite concentrations is an important consideration in translational studies.

Importantly, in our study, we directly measured creatine levels in the peritoneal fluid of patients with endometriosis and found that they were approximately 150–400 µM, suggesting substantial local accumulation within the peritoneal microenvironment [1]. Based on these observations, we designed a concentration gradient of 0, 10, 100, and 1000 µM creatine in our in vitro experiments to model a range of potential exposure conditions that cells may encounter in vivo. For mechanistic analyses, we selected 1000 µM creatine as a high‐exposure, supraphysiological condition intended to elicit robust and reproducible cellular responses under defined experimental settings. We agree that this concentration exceeds the creatine levels directly measured in patient peritoneal fluid and should not be interpreted as fully physiologically representative. Such exposure paradigms are nevertheless commonly used in exploratory mechanistic studies to maximize detectable biological effects within experimentally tractable timeframes.

For the in vivo mouse experiments, a relatively high creatine concentration was administered to achieve transient local enrichment and ensure sufficient exposure within a relatively short experimental timeframe. Such dosing strategies are commonly employed in metabolic and pharmacological studies to account for differences in distribution and metabolism between experimental models and human physiology. We agree that future pharmacokinetic analyses examining local creatine concentrations within the murine peritoneal cavity would further strengthen the translational interpretation of these findings.

Importantly, our conclusions do not rely solely on exogenous creatine supplementation. Our metabolomic and transcriptomic analyses further demonstrated increased endogenous creatine synthesis in ectopic endometrial stromal cells, including elevated expression of enzymes involved in creatine metabolism. These findings suggest that creatine accumulation represents an intrinsic metabolic feature of endometriotic lesions, rather than merely an artifact of experimental supplementation.

Nevertheless, we acknowledge that future studies incorporating detailed pharmacokinetic measurements and broader concentration‐response analyses will be important for further defining the physiological relevance of creatine exposure in endometriosis.

## 2 Evidence for the Interaction Between Creatine and PrP

The Comment suggests that additional biochemical validation would be necessary to confirm a direct molecular interaction between creatine and prion protein (PrP). We appreciate this point and would like to clarify the experimental framework used in our study.

In our work, we first applied the drug affinity‐responsive target stability (DARTS) assay coupled with mass spectrometry analysis to identify candidate creatine‐binding proteins^1^. This unbiased screening approach identified PrP as a candidate functional target protein. To further explore the potential interaction interface, we performed molecular docking analysis (AutoDock), which predicted plausible binding sites for creatine within the functional region of PrP. Beyond target identification and computational prediction, we conducted functional validation experiments demonstrating that creatine is associated with modulation of PrP‐linked iron metabolism. Specifically, creatine treatment reduced the PrP‐mediated conversion of Fe^3^^+^ to Fe^2^^+^, leading to decreased iron uptake and attenuation of ferroptosis in endometrial stromal cells. These functional observations provide mechanistic support consistent with a PrP‐associated regulation of cellular iron homeostasis upon creatine exposure.

We agree that biochemical approaches using purified proteins, such as direct binding affinity measurements or enzymatic activity assays, could further strengthen the characterization of this interaction. Importantly, we have already acknowledged this limitation in the Discussion section of our original article^1^. As noted there, the membrane localization of PrP, either on the cell surface or within endosomes, imposes technical constraints on precisely evaluating its catalytic activity in vitro. We therefore suggested that future studies should aim to map the precise creatine‐binding site on PrP, verify its alignment with the predicted active region, and determine how creatine binding may influence PrP conformation and functionality.

Taken together, our study integrates target identification (DARTS), structural prediction (molecular docking), and functional validation experiments, providing converging evidence consistent with a creatine–PrP interaction axis in regulating ferroptosis resistance. We agree that future biochemical studies will further refine the molecular details of this interaction.

## 3 Interpretation of Human Sample Data

The Comment notes that the human cohort size in our study may limit clinical extrapolation and that potential confounding factors were not fully controlled. We appreciate this observation and agree that large, well‐controlled cohorts are essential for establishing reliable clinical biomarkers.

In our study, the analysis of human samples was primarily intended to provide translational support for the mechanistic findings obtained from cellular and experimental models rather than to establish a definitive clinical biomarker. Despite the moderate cohort size, we consistently observed elevated creatine levels in both peritoneal fluid and ectopic endometrial stromal cells from patients with endometriosis. Importantly, we applied several inclusion criteria to minimize potential confounding factors. Participants in our cohort did not receive creatine supplementation, had normal liver and renal function, and samples were collected during the proliferative or secretory phases of the menstrual cycle, thereby reducing variability associated with metabolic status or hormonal fluctuations. We fully acknowledge that additional variables, including dietary protein intake, disease stage at the time of sampling, concurrent medication use, systemic inflammatory burden, and body mass index (BMI), were not formally incorporated into our analysis. These factors may influence peritoneal and tissue creatine levels and should be considered in future studies when interpreting the variability and translational implications of these findings.

In addition to our clinical samples, we further examined publicly available transcriptomic datasets from the Gene Expression Omnibus (GEO) to validate the metabolic alterations related to creatine metabolism observed in our study. These independent datasets provided additional support for the dysregulation of creatine‐related pathways in endometriosis.

Importantly, the human data were not used as the sole basis for causal inference, but rather as supportive evidence for the metabolic alterations identified through mechanistic experiments. We agree that future studies with larger patient cohorts and more detailed clinical stratification will be valuable for further validating the role of creatine metabolism in endometriosis and exploring its potential clinical relevance.

## 4 Causality Versus Association

The Comment raises an important conceptual question regarding whether creatine acts as a causal driver of ferroptosis resistance or is merely associated with the inflammatory microenvironment of endometriosis.

In our study, several lines of evidence support a contributory role of creatine in ferroptosis resistance in endometriosis. First, we observed significant creatine accumulation in both the peritoneal fluid and ectopic lesions of patients with endometriosis, accompanied by increased expression of enzymes involved in endogenous creatine synthesis, suggesting that creatine metabolism is actively altered within the disease microenvironment. Second, functional experiments demonstrated that exogenous creatine supplementation directly modulates ferroptosis susceptibility in endometrial stromal cells under iron‐rich conditions. Creatine treatment reduced intracellular Fe^2^^+^ accumulation and attenuated ferroptotic cell death, indicating that creatine can directly influence iron metabolism and ferroptosis sensitivity. Third, in vivo experiments further showed that creatine administration promoted the progression of endometriotic lesions in a mouse model, supporting the pathological relevance of creatine in the development of endometriosis.

Taken together, these findings from clinical observations, functional cellular assays, and in vivo models support creatine as a modulatory contributor to ferroptosis resistance in endometriotic lesions, rather than merely reflecting a passive association with the inflammatory microenvironment.

Nevertheless, we agree that additional experimental approaches, such as reducing intracellular creatine levels by manipulating creatine synthesis enzymes or creatine transport systems, would provide further mechanistic insight and help refine the causal framework of this pathway. These experiments represent important directions for future investigation.

## 5 Conclusion

We thank Dr. Machado for the constructive discussion of our work. We believe that the points raised in the Comment highlight valuable directions for future investigation while remaining consistent with the central findings of our study. Our results demonstrate that creatine accumulation in endometriotic stromal cells contributes to ferroptosis resistance through modulation of PrP‐dependent iron metabolism, providing a new perspective on the metabolic regulation of endometriosis. Importantly, our study is intended to provide translational support for mechanistic studies in endometriosis rather than to establish clinical biomarkers or immediate diagnostic utility. We hope that this exchange will stimulate further research into the role of creatine metabolism in the pathogenesis of endometriosis.

## Conflicts of Interest

The authors declare no conflicts of interest.

## Data Availability

Data sharing not applicable to this article as no datasets were generated or analyzed during the current study.
